# Myopia: a growing epidemic

**Published:** 2019-05-13

**Authors:** Judith Flanagan, Tim Fricke, Priya Morjaria, Sumrana Yasmin

**Affiliations:** 1 Senior Scientist: Brien Holden Vision Institute, Sydney, Australia.; 2 Senior Research Fellow and Paediatric Optometrist: Brien Holden Vision Institute, Sydney, Australia.; 3 Research Fellow: Department of Clinical Research, London School of Hygiene and Tropical Medicine, International Centre for Eye Health, London, UK.; 4 Regional Director: South East Asia and Eastern Mediterranean, Brien Holden Vision Institute, Islamabad, Pakistan.

**Myopia is a serious and growing problem that will affect low- or middle-income countries as they become more urbanised – especially when educational demands increase.**

Evidence from various countries, age groups, and ethnicities^1^ indicates that myopia, defined as refractive error ≤-0.50 D in the least myopic eye, currently affects approximately 28% of the global population.^2^ In the highly developed urban areas of East and South East Asia, as many as 90% of school leavers have myopia.^2^,^3^ In Europe and North America, 30–50% of school leavers are affected and, in sub-Saharan Africa, myopia affects 5–15% of these children.^2^,^4^

Uncorrected myopia is the leading cause of blindness worldwide. In 2015, there were 124 million people around the world with moderate or severe vision impairment (MSVI) or blindness due to uncorrected refractive error. The other leading causes were cataract (66 million people) age-related macular degeneration (10 million people), glaucoma (7 million), diabetic retinopathy (3 million) and other (or unidentified) causes (37 million).^5^

There are two main ways myopia can cause visual impairment. The first is via un- or under-corrected refractive error. Distance vision impairment can result when a person with myopia is unable to get appropriate spectacles or contact lenses or have them updated as needed. Second, increasing myopia is associated with increasing prevalence of visual impairment from complications that cause irreversible visual loss, including glaucoma and vitreo-retinal diseases such as myopic macular degeneration and retinal detachment.

Evidence consistently suggests that the global prevalence of myopia is increasing,^2^ with the rate of increase being particularly alarming in many Asian countries.^6^ Holden et al. (2016) predicted that the global prevalence of myopia will rise from 28% (2 billion people) in 2010 to 50% (5 billion people) in 2050.^2^ The same study predicted that the global prevalence of high myopia will rise from 4% (227 million people) in 2010 to 10% (938 million) in 2050.^2^

Environmental factors and lifestyle changes, such as increased time indoors (related to increased educational demands^7^), increased use of electronic devices and decreased time spent outside are highly associated with the increased prevalence of myopia.^3^ There are also reports implicating factors such as town planning (the design of our built environment) and diet (higher saturated fat and cholesterol intake).^8^

**Figure:**
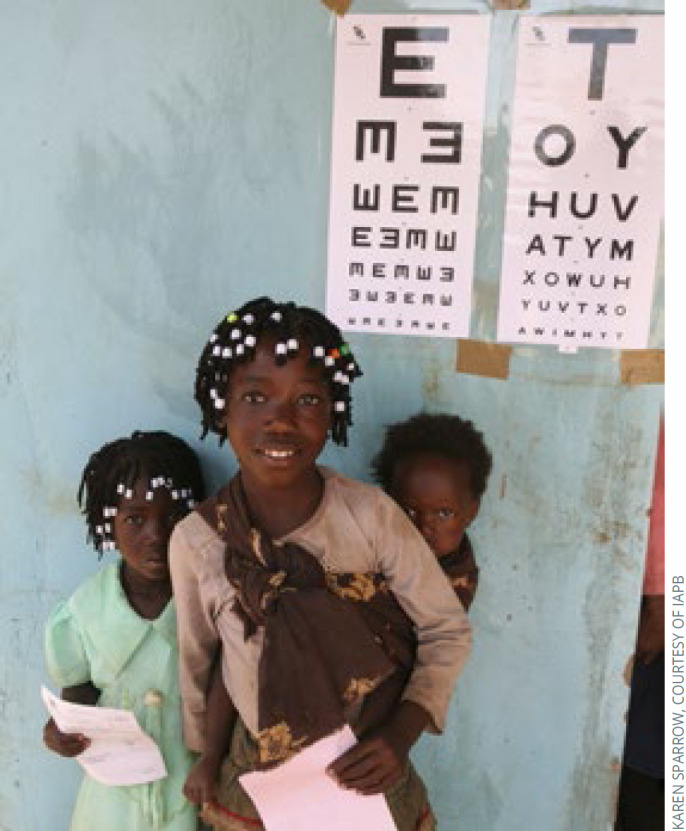
Children waiting for an eye examination. ZAMBIA

## Changing demographics

The groups of people affected by myopia (or the demographics of myopia) appears to be changing in two ways that are important in the link between myopia and visual impairment^2^:

1. As countries develop and people become more urbanised, the myopia epidemic will increasingly affect areas with fewer resources and with health systems that are less ready to deal with myopia and its complications.
2. Even though myopia will initially only affect children, the fact that it is a life-long condition means that it will ‘spread’ to all age groups over the next several decades.

People who live in a low-income setting will be less likely to have access to adequate optical correction and the health care systems needed to adequately manage the complications associated with myopia (p. 11).^9^,^10^
